# P2X7 receptor: the regulator of glioma tumor development and survival

**DOI:** 10.1007/s11302-021-09834-2

**Published:** 2021-12-29

**Authors:** Damian Matyśniak, Vira Chumak, Natalia Nowak, Artur Kukla, Lilya Lehka, Magdalena Oslislok, Paweł Pomorski

**Affiliations:** 1 Laboratory of Molecular Basis of Cell Motility, Nencki Institute of Experimental Biology of Polish Academy of Sciences, 3 Pasteur Str., 02-093 Warsaw, Poland; 2 Regenerative Medicine Department, Medical University of Warsaw, Warsaw, Poland; 3 Laboratory of Imaging Tissue Structure and Function, Nencki Institute of Experimental Biology of Polish Academy of Sciences, Warsaw, Poland; 4 Silesian University of Technology, Gliwice, Poland; 5 Institute of Cell Biology, National Academy of Sciences of Ukraine, Lviv, Ukraine; 6 Department of Embryology, Faculty of Biology, University of Warsaw, Warsaw, Poland

**Keywords:** P2X7, Glioma, Cell survival, ROS signaling

## Abstract

P2X7 is an ionotropic nucleotide receptor, forming the cation channel upon ATP stimulation. It can also function as a large membrane pore as well as transmit ATP-dependent signal without forming a channel at all. P2X7 activity in somatic cells is well-known, but remains poorly studied in glioma tumors. The current paper presents the comprehensive study of P2X7 activity in C6 and glioma cell line showing the wide range of effects the receptor has on glioma biology. We observed that P2X7 stimulation boosts glioma cell proliferation and increases cell viability. P2X7 activation promoted cell adhesion, mitochondria depolarization, and reactive oxygen species overproduction in C6 cells. P2X7 receptor also influenced glioma tumor growth in vivo via activation of pro-survival signaling pathways and ATP release. Treatment with Brilliant Blue G, a selective P2X7 antagonist, effectively inhibited glioma tumor development; decreased the expression of negative prognostic cancer markers pro-survival and epithelial-mesenchymal transition (EMT)-related proteins; and modulated the immune response toward glioma tumor in vivo. Finally, pathway-specific enrichment analysis of the microarray data from human patients also showed an upregulation of P2X7 receptor in gliomas from grades I to III. The presented results shed more light on the role of P2X7 receptor in the biology of this disease.

**Supplementary Information:**

The online version contains supplementary material available at 10.1007/s11302-021-09834-2.

## Introduction

P2X7 purinoceptor belongs to the subfamily of ligand (ATP)-gated ionotropic P2X receptors and is a trimeric ion channel that modulates transmembrane calcium, sodium, and potassium ion movement [1]. P2X7 plays an important role in inflammation, immunity, bone homeostasis, neurological function, and neoplasia [2]. The best characterized activity of P2X7 is its role in ATP-induced IL-1β release from macrophages and microglia [3]. Extracellular ATP induces P2X7 activation and channel opening for Ca^2+^ and Na^+^ influx and K^+^ efflux [4, 5]. Sustained stimulation of P2X7 with higher ATP doses or repeated stimulation with sequential ATP pulses induces the formation of a large pore permeable for molecules up to 900 Da [6, 7]. This ability is associated with an intact, non-mutated C-terminal domain of the receptor [8]. The ATP-induced pore formation leads to cell death [9] in various types of cells: macrophages [10, 11], microglial cells [12], neurons [13], and some cancer cells [14, 15].

P2X7 is responsible for proliferation [16], migration, and invasiveness of many cancer cells [16, 17]. The receptor is expressed in human brain tumors including malignant gliomas [18–21]. However, the data about P2X7 role in this type of tumors is highly controversial. On one hand, a P2X7-dependent rise in intracellular calcium and ATP cytotoxicity was shown in murine astrocyte-derived glioma cell GL261 [22]. Other authors demonstrated that P2X7 plays a pivotal role in inhibiting glioma stem cell growth [19]. On the other hand, activation of P2X7 in gliomas was associated with increases in intracellular mobilization of Ca^2+^, enhanced mobility of cells, and elevated cellular expression of factors promoting inflammation and tumor vascularization [23]. P2X7 activation by extracellular ATP induces the secretion of more ATP and it may be the part of a self-amplifying feed-forward mechanism ensuring continuous activation of P2X7 [24]. In glioblastoma cells, as in some other cancer cells, Ca^2+^ channels are involved in uncontrolled proliferation, enhanced migration and invasion, sustained angiogenesis, and abnormal cell death [25, 26]. The abovementioned data demonstrate that P2X7 receptors stimulate glioma development and aggressiveness. However, the responsible pathways remain to be revealed. What is more, the availability of a large number of P2X7 antagonists makes the receptor a particularly attractive target for new therapies; thus, its role in glioma biology is undoubtedly worth further investigation [17, 26–30].

In the current study, we demonstrated that the activated P2X7 receptor is responsible for massive ATP release, and an increase of calcium signal in glioma C6 cells. P2X7 activation stimulated C6 glioma cell proliferation and survival in vitro and increased expression of pro-survival proteins, such as HSPA1, HSPA5 chaperones, and CD133 as well as p38 MAPK and AKT kinase phosphorylation. Moreover, inhibition of P2X7 reduced tumor mass and tumor development in vivo with a simultaneous decrease of cancer-associated pro-survival protein expression. It seems that the P2X7 receptor can be engaged in shaping of glioma tumor microenvironment through modulation of inflammation marker profile, epithelial-to-mesenchymal transition components, and extracellular ATP release in C6 glioma cells. This paper interprets the data obtained from a C6 rat model and bioinformatical analysis of P2X7 expression in human glioma samples to bring some new insight on the biology of this disease.

## Materials and methods

### Cell culture

Rat glioma cell line C6 and human glioma line U-138 MG obtained from ATCC (C6) and DSMZ (U-138 MG) were cultured in DMEM high-glucose medium (4.5 g/l glucose, Thermo Fisher Scientific Inc., USA), supplemented with 10% heat-inactivated FBS (Thermo Fisher Scientific Inc., USA). Cells were grown in standard conditions (37 °C, 5% CO_2_, 95% humidity). Cell lines were routinely tested to exclude mycoplasma contamination using PCR Mycoplasma Detection Kit (Applied Biological Materials Inc., Canada), according to the manufacturer’s instruction. Before BzATP (Jena Bioscience, Germany) treatment, C6 cells were cultured in serum-free DMEM (Lonza, Switzerland) with N-2 supplement (Gibco™, USA) for 24 h.

#### P2X7 RNA interference

Three target-specific RNAi (small interfering RNA) complementary to rat P2X7 transcript (sc-108056) and control non-silencing RNAi were obtained from Santa Cruz Biotechnology, Inc. (USA). C6 glioma cells were seeded onto a Petri dish to obtain 80% confluence. After 24 h of incubation in serum-containing medium, the cells were transfected using TransIT-X2 transfection reagent (Mirus Bio LLC, USA) according to the manufacturer’s recommendations. After 72 h of incubation with RNAi, the medium was changed to fresh, and after 24 h of recovery cells with a decreased level of P2X7 protein were used for experiments.

#### Cell proliferation after P2X7 downregulation

C6 cells after P2X7 silencing were seeded onto 96 wells (5 × 10^3^/well). At specified time points (24, 46, 72, 96 h), MTS (3-(4, 5-dimethylthiazol-2-yl)-5-(3-carboxymethoxy-phenyl)-2-(4-sulfophenyl)-2H-tetrazoli-um) metabolic activity assay was performed to measure cell viability and growth according to the manufacturer’s instructions. The absorbance was read using Sunrise plate reader (Tecan Trading AG, Switzerland) at 490 nm.

#### Calcium signal measurements

Calcium signal measurements were performed as described previously [31] using Fura-2 AM ratiometry [32]. Briefly, glioma cells (1.5 × 10^4^) were seeded onto rectangular glass coverslips in 35-mm dishes the day before the experiment. Cells (50–70% confluent) were loaded with 50 μM Fura-2 AM (Thermo Fisher Scientific Inc., USA) in serum-depleted culture medium for 30 min at 37 °C in a humidified atmosphere of 95% air and 5% CO_2_. The cells were then washed twice with the solution composed of 5 mM KCl, 1 mM MgCl_2_, 0.5 mM Na_2_HPO_4_, 25 mM HEPES, 130 mM NaCl, 1 mM pyruvate, 5 mM _D_-glucose, and 0.1 mM CaCl_2_, at pH 7.4, and the coverslips were mounted in a cuvette filled with 1.5 ml Ca^2+^-containing assay solution (as above but with 2 mM CaCl_2_) and maintained at RT in a RF5001PC spectrofluorometer (Shimadzu Corp., Japan). Fluorescence was measured at 510 nm with excitation at 340 and 380 nm every 1.4 s for at least 300 s. Excitation and emission slit widths were 2.5 nm and 20.0 nm, respectively. Calcium signal was evaluated as changes in F340/F380 fluorescence intensity ratio. Specific treatments were applied after at least 60 s from the start of the measurement to alleviate the effect of dye bleaching. The cells were treated with 300 μM BzATP (Jena Bioscience, Germany).

### Flow cytometric ROS measurement

C6 cells were maintained in serum-free DMEM (Lonza, Switzerland) with N-2 supplement (Gibco™, USA) for 24 h before the experiment. C6 cells were treated with 100 μM BzATP for 1 h, washed twice with PBS, and stained with 10 μM 2',7'-dichlorofluorescin diacetate (DCF-DA) (Sigma Aldrich, USA) for 30 min at 37 °C. The DCF fluorescence was measured using the Guava® easyCyte 8HT Benchtop Flow Cytometer (Merk Millipore, Germany) using a green filter 525/30.

#### Mitochondria staining using MitoTrackerCMX ROS

C6 cells were maintained in serum-free DMEM (Lonza, Switzerland) with N-2 supplement (Gibco™, USA) for 24 h before the experiment. C6 cells were treated with 100 μM BzATP for 1 h, washed twice with 1 × PBS, and stained with 500 nM MitoTrackerCMX ROS (Thermo Fisher Scientific, USA) for 30 min at 37 °C. Live cell imaging was performed using the Zeiss LSM 780 microscope with a × 40 water objective lens 1.2 NA (Carl Zeiss Microscopy GmbH, Germany) equipped with an environmental chamber, using the appropriate excitation laser and detection range.

### JC‐1 probe: analysis of mitochondrial membrane potential (Δψm)

C6 cells were maintained in serum-free DMEM (Lonza, Switzerland) with N-2 supplement (Gibco™, USA) for 24 h before the experiment. C6 cells were treated with 100 μM BzATP for 1 h, washed twice with 1 × PBS, and stained with 5 μM JC-1 (Invitrogen, USA) for 30 min at 37 °C. Relative degree of mitochondrial polarization was quantified by measuring the red‐shifted JC‐1 aggregates (yellow filter 583/26) and the green‐shifted monomers (green filter 525/30) ratio using the Guava® easyCyte 8HT Benchtop Flow Cytometer (Merk Millipore, Germany).

### Rapid cell adhesion assay

The 96-well cell culture plates (CytoOne, USA) were coated with collagen I (50 μg/ml) (Sigma Aldrich, USA), collagen IV (50 μg/ml) (Sigma Aldrich, USA), or fibronectin (10 μg/ml) (Sigma Aldrich, USA) overnight at 2–8 °C and dried in a sterile tissue culture hood for an hour. C6 cells, cultured in serum-free DMEM with N-2 supplement (24 h before the experiment), were treated with 100 μM BzATP for 24 h and trypsinized and the cell suspension (1 × 10^6^ cells/ml) was prepared. One hundred microliters of such cell suspension was seeded onto previously coated 96-well cell culture plates. The cells were incubated for 20 min at 37 °C and washed with 1 × PBS to remove non-attached cells. Next, the cells were fixed by incubation with ice-cold methanol for 20 min, and stained with 0.1% (w/v) crystal violet for 10 min at room temperature. Then, the wells were washed 3 times with tap water and the dye was solubilized in 100 μl 10% (v/v) acetic acid. The absorbance was measured at 570 nm using Sunrise plate reader (Tecan Trading AG, Switzerland).

### Cell invasion transwell assay

Eight-micrometer pore-size transwell cell culture chambers (Corning, USA) were coated with 30 μl of Corning® Matrigel® Matrix (Corning, USA) and allowed to polymerize at 37 °C in a humidified incubator for 1 h. Then, 10 × 10^4^ of LN-229 or U-251 cells resuspended in 200 μl serum-free DMEM with 2 μM KN-62 was seeded per well to the upper chamber. The bottom chamber contained 100% FBS. Before the plating into transwell chambers, cells were cultured in serum-free DMEM for 24 h. After 24 h, inserts were washed in PBS, fixed for 10 min using ice-cold methanol, and stained with 0.1% crystal violet. Non-migration cells in the upper chamber were removed by wiping the upper side of the membrane with a cotton swab. Microphotographs of the invaded cells at the bottom of the insert were obtained using an Olympus (Olympus, Japan) and the cell numbers from five random fields were quantified using the ImageJ software.

### Crystal violet assay

A total of 10 × 10^4^ of C6, LN-229, or U-251 cells was seeded per well and after 24 h cells were treated with 100 nM BBG, 2 μM KN-62, 200 μM of BCNU, and 600 μM of TMZ. Before the seeding into 96-well plates, cells were cultured in serum-free DMEM for 24 h. After 24 h, attached cells were washed in PBS, fixed for 10 min using ice-cold methanol, and stained with 0.1% crystal violet. Then, 50 μl solubilization solution (10% acetic acid, 0.1% Tween 20) was added and incubated for 1 h at RT. The absorbance was measured at 570 nm using the Sunrise plate reader (Tecan Trading AG, Switzerland).

### Fluorescence-activated cell sorting (FACS) analysis of CD68 and FOXP3 cellular markers

Tumor tissue was incubated with collagenase (2 mg/ml in DMEM) (Thermo Fisher Scientific Inc., USA) for 1 h at 37 °C with the following dissociation using gentleMACS™ Dissociator (Miltenyi Biotec, USA). For staining of FOXP3 + and CD68 + cells, we used anti-FOXP3 antibody conjugated to Alexa Fluor 488 and anti-CD68 antibody conjugated to APC (BioLegend, USA), respectively. To exclude non-specific antibody interaction, rat isotypic IgG control (BioLegend, USA) was used. Staining was performed in PBS containing 1% BSA (BioShop, Canada). Antibodies were added at concentrations recommended by the manufacturer. All samples were incubated for 30 min at 37 °C, washed, and analyzed using Guava® easyCyte 8HT Benchtop Flow Cytometer (Merck Millipore, Germany).

### Protein extraction and Western blot analysis

Glioma cells (1 × 10^6^) were lysed using RIPA buffer (Thermo Fisher Scientific Inc., USA) supplemented with cOmplete™ Protease Inhibitor Cocktail (Roche Applied Science, Switzerland). The protein concentration of whole cell extracts was measured using the Protein Assay Kit (Bio-Rad Laboratories, Inc., USA). Tumor samples were homogenized in RIPA buffer supplemented with cOmplete™ Protease Inhibitor Cocktail (Roche Applied Science, Switzerland) in a mass ratio 1:9. Samples were resolved using SDS-PAGE in 8% acrylamide gel (30 μg of protein from cells or 5 μl of tumor homogenate per well) and blotted onto nitrocellulose membrane using a Trans-BlotTurbo transfer system (Bio-Rad Laboratories Inc., USA). Membranes were blocked in fat-free 5% milk in TTBS (0.25 M Tris–HCl, pH 7.5, 0.15 M NaCl, and 0.1% Tween-20) and incubated overnight with primary antibody at 4 °C. Antibodies used for the immunodetection are described in Supplementary Table S1. The signal from both primary antibodies was visualized using the Pierce™ ECL Western Blotting Substrate (Thermo Fisher Scientific Inc., USA). Immunodetected proteins were imaged on X-ray film (Kodak, USA); densitometry was performed using Fiji distribution [33] of ImageJ2 [34] and integrated density normalized to β-tubulin band.

### Gelatin zymography

Tumor homogenates were prepared in a non-reducing sample buffer (125 mM Tris–HCl, 4% SDS, 20% glycerol, 0.01% bromophenol blue) in a mass ratio 1:9 and separated in 7.5% acrylamide gel containing 10% of sodium dodecyl sulfate (BioShop, Canada) and 4 mg/ml gelatin (BioShop, Canada). After the electrophoresis, gel was washed twice in washing buffer (50 mM Tris–HCl, 5 mM CaCl_2_, 1 μM ZnCl_2_, 2.5% Triton X-100, pH 7.5). Then, the gel was incubated overnight at 37 °C in incubation buffer (50 mM Tris–HCl, 5 mM CaCl_2_, 1 µM ZnCl_2_, 1% Triton X-100, pH 7.5) for the development of zymolytic bands. Protease bands were detected by the absence of Coomassie Brilliant Blue staining of the digested gelatin. Gel’s images were analyzed using Fiji distribution as described above.

### *In vivo *model of C6 glioma tumor treatment

All protocols and procedures were approved by the Institutional Animal Care and Use Committee of Institute of Cell Biology, NASU (Lviv, Ukraine). One million of C6 glioma cells suspended in 100 µl of Matrigel (Corning Inc., USA) were inoculated subcutaneously into the right limb of 3-month-old C57/BL6J male mice. The successful C6 rat glioma xenotransplantation into the immunocompetent mice was described previously [35]. Palpable tumors were developed and we started subcutaneous administration of Brilliant Blue G (BBG) (Sigma Aldrich, USA) on the 10th day after C6 cell inoculation. One hundred milligrams of BBG per 1 kg of body weight was administered every 48 h. Animals from the control group were injected with an equivalent volume of PBS. The cumulative dose of BBG was 400 mg/kg of body weight. At the end of the experiment, mice were euthanized using cervical dislocation preceded with isoflurane anesthesia. The tumors were isolated, the tumor mass was immediately recorded, and the tumor tissues/peripheral blood serum proceeded accordingly to each experimental procedure.

### Blood sampling and serum preparation

Peripheral blood (PB) was collected immediately after euthanasia using the cardiac puncture technique. Next, the blood was allowed to clot for 30 min at 37 °C and was centrifuged at 4 °C for 10 min at 2000 × *g* to remove the clot. Supernatant (serum) was collected, aliquoted, and kept at − 80 °C until analysis.

#### ATP level measurement

ATP amount was measured in C6 cell-cultured medium, in PB serum of experimental animals, and in tumor tissue. After C6 cell treatment with 100 μM BzATP for 24 h, conditioned medium was collected and proceeded as described below. Tumor samples were homogenized in buffer A (25 mM Tris–HCl, 150 mM KCl, 2 mM EDTA, 0.1% EDTA, 10 mM KH_2_PO_4_, 0.1 mM MgCl_2_, pH 7.4) in a mass ratio 1:9. ATP extraction from conditioned medium, PB serum, and tumor homogenates was performed using 1.5% TCA (Sigma Aldrich, USA). Next, pH was adjusted to 7.5–7.8 using 1 M Tris-OH buffer pH 10. For ATP measurement, 1 μl of medium, serum, or clarified tumor homogenate was diluted in 999 μl of buffer A. In total, 100 μl of sample was used for each assay. All samples were diluted to the same final concentration within each type of experiment. The measurement procedures were performed according to the manufacturer’s protocol using ENLITEN® ATP Assay System (Promega, USA). ATP levels were evaluated by luminescence measurement using the Infinite M1000 PRO plate reader (Tecan Trading AG, Switzerland) and expressed in relative light units (RLU).

#### Immunofluorescence and histopathological staining

Isolated tumors were fixed immediately with freshly prepared 4% paraformaldehyde (Santa Cruz Biotechnology, USA) for 24 h at 4 °C with the following overnight incubation in 30% (w/v) sucrose solution and frozen in isopentane cooled by liquid nitrogen. Frozen tumor tissues were cut using Leica CM 1950 cryostat (Leica Biosystems, Germany) into 20-µm-thick sections. The tumor sections were fixed onto slides with cold 4% paraformaldehyde for 10 min, washed three times with PBS, and permeabilized using 0.05% Triton X-100 solution in PBS for 30 min at room temperature. Unspecific binding was blocked with 5% (v/v) horse serum in PBS. Anti-P2X7 antibody (Alomone Labs, Israel) was diluted as recommended in the manufacturer’s instruction and incubated with tissue overnight at 4 °C. The next day, the slides were washed with PBS and tumor sections were incubated with anti-rabbit Alexa Fluor 488-conjugated antibody (Invitrogen, USA) for 2 h at room temperature. Tumor slices were mounted in a Vectashield mounting medium with DAPI (Vector Laboratories, USA) and the samples were observed using the Zeiss LSM 780 confocal microscope with × 63 oil objective lens 1.4 NA (Carl Zeiss Microscopy GmbH, Germany) using an appropriate excitation laser and detection range. For the histopathological examination, frozen tumors were fixed with freshly prepared 4% paraformaldehyde (Santa Cruz Biotechnology, USA) for 24 h at 4 °C and then processed using a routine wax-embedding procedure for histological examinations. Three-micrometer-thick sections were stained with hematoxylin and eosin (Sigma Aldrich, USA).

#### Cytokine array

For the evaluation of cytokine levels in PB serum, we used Mouse Cytokine Antibody Array — Membrane (ab133999, Abcam, UK) — and in tumors, Rat Cytokine Antibody Array — Membrane (ab133992, Abcam, UK). The tumor tissues were homogenized in the same way as for Western blot using the provided 2 × cell lysis buffer, properly diluted 1:1 with deionized H_2_O, and supplemented with cOmplete™ Protease Inhibitor Cocktail (Roche, Switzerland). One microliter of plasma/proper volume of tumor tissue lysate was diluted in 1 ml of 1 × blocking buffer, loaded onto the membrane, and incubated overnight at 4 °C. All tumor samples were diluted to the same final concentration of total lysed protein and processed according to the manufacturer’s protocol. Cytokine spot immunodetection was performed on X-ray film (Kodak, USA). Signal density was analyzed with the ImageJ software protein array analyzer plug-in. The fibril spots containing the highest protein amount (500 ng) were analyzed by densitometry using the Gilles Carpentier’s dot-blot-analyzer macro (written by Gilles Carpentier, 2008; the macro is available at http://rsb.info.nih.gov/ij/macros/toolsets/Dot%20Blot%20Analyzer.txt and more information can be found at http://image.bio.methods.free.fr/dotblot.html) written for ImageJ [34].

#### Colorimetric superoxide dismutase (SOD) activity quantification

SOD activity measurement was performed using Superoxide Dismutase (SOD) Activity Assay Kit (BioVision, USA). Ten milligrams of tumor fragments was weighed, homogenized in ice-cold 0.1 M Tris–HCl, pH 7.4 containing 0.5% Triton X-100, 10 mM DTT, and 0.1 mg/ml PMSF, and centrifuged at 12,000 × *g* for 10 min at 4 °C to discard the cell debris. SOD activity measurement and SOD activity calculation were performed according to the manufacturer’s protocol. The absorbance was measured at 450 nm using Infinite M1000 PRO plate reader (Tecan Trading AG, Switzerland).

### Oxidized (GSSG)/reduced (GSH) glutathione ratio evaluation

Every 10 mg of tumor tissue was disintegrated in 250 μl ice-cold 100 mM phosphate buffer, pH 7. Then, 250 μl of 1.5% TCA was added, incubated for 10 min, and the samples were centrifuged at 12,000 × *g* for 10 min at 4 °C. Reaction mixture was prepared according to the protocol of Glutathione Colorimetric Detection Kit (Invitrogen, USA). The absorbance was measured at 405 nm using Infinite M1000 PRO plate reader (Tecan Trading AG, Switzerland). The GSSG/GSH ratio was calculated using the following equations:

$$\begin{array}{c}ratioGSSG/GS{H}_{control}=\left(2\cdot gss{g}_{contr}/\left(gs{h}_{contr}+\left(2\cdot gss{g}_{contr}\right)\right)\right)\cdot 100\%\\ ratioGSSG/GS{H}_{exp}=\left(2\cdot gss{g}_{exp}/\left(gs{h}_{exp}+\left(2\cdot gss{g}_{exp}\right)\right)\right)\cdot 100\%\end{array}$$

### Bioinformatical data analysis

The dataset for analysis was downloaded from The Cancer Genome Atlas Glioblastoma Multiforme (TCGA-GBM, https://www.cancer.gov/tcga) using RTCGA package family in R software environment (version 3.6.1 by RStudio Team (2020); RStudio: Integrated Development for R. RStudio, PBC, Boston, MA URL http://www.rstudio.com/). *TCGA’s Study of Glioblastoma Multiforme* was originally published by the National Cancer Institute. We decided to select data from patients whose survival time was greater than 100 and less than 1000 days to avoid uncertain results (*n* = 126). Using maximally selected rank statistics provided by the survminer package, the optimal cut point for continuous variables was determined. Based on P2X7 expression, we estimated the optimal cut point and divided the data into two groups: low- and high-P2X7 expression. The Kaplan–Meier plots were created for both groups.

The dataset containing information about gene expression in astrocytoma tumor tissues (*n* = 17) and in normal brain tissue (*n* = 4) was downloaded from NCBI database (https://www.ncbi.nlm.nih.gov/geo/query/acc.cgi?acc=GSE19728&fbclid=IwAR251_GjISIUTUnnxP7WnlRN21e7WM_OqgG0Y3FYolHU6qxsmkpmG WHvVL4). A boxplot was created to demonstrate an expression of P2X7 gene in astrocytomas of WHO grades I–IV and in normal tissues. Quality control and analysis were made in R software environment.

### Statistical data analysis

Data are presented as fold changes compared to control or as mean ± SD. Plots were generated using Matplotlib 3.1.0 Python library (Hunter, 2007) unless otherwise stated. Asterisks represent statistically significant differences in comparison to the control. *n* refers to the number of independent biological repetitions, at least three technical repetitions each. Statistical analysis was performed on the raw data using SigmaPlot 12.3 (Systat Software Inc., USA). To assess the differences between the treatments and the control, paired *t*-Student test or repeated-measures one-way ANOVA followed by Bonferroni post hoc test was used. Data normality and variance equality were assessed with Shapiro–Wilk test and Levene’s test, respectively. Differences were considered as statistically significant at *p* < 0.05.

### Raw data accessibility

Raw data is available on request from the authors.

## Results

### P2X7 receptor is active in C6 cells *in vitro*

**Fig. 1:**
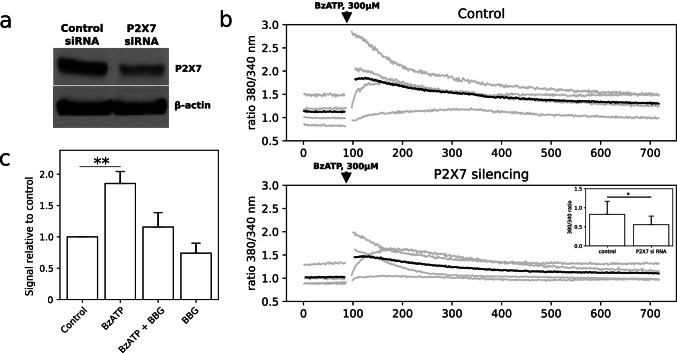
Characterization of P2X7 receptor in glioma C6 cells in vitro. **a** Western blot analysis of P2X7 protein expression in C6 cells after P2X7 silencing for 72 h (*n* = 3). **b** Calcium signal level in glioma C6 cells after P2X7 downregulation and BzATP stimulation (300 µM) (*n* = 4). Gray lines represent the signals from the biological replicates; black lines represent the signal averaged among the individual replicates. Insert shows effect of P2X7 downregulation on the signal strength, one-way *t*-test: *P ≤ 0.05. **c** Measurement of bioluminescence originated from released ATP after P2X7 activation/inhibition in C6 cells (*n* = 3). The statistical significance of the differences was determined with one-way ANOVA with Bonferroni post hoc test: *P ≤ 0.05, **P ≤ 0.01, ***P ≤ 0.001 vs. control group

To study the activity of P2X7 receptor in rat glioma C6 cells in vitro, we used BzATP (2' (3')-O-(4-benzoylbenzoyl) adenosine-5'-triphosphate) [36] and a highly potent antagonist Brilliant Blue G (BBG) [29, 37]. P2X7 receptor stimulation with 300 μM BzATP for 5 min resulted in the increase of calcium signal in C6 cells. To confirm that calcium signal after BzATP stimulation originated from P2X7 receptor, we used cells with RNAi-mediated downregulation of P2X7 (Fig. 1a). Upon BzATP stimulation, cells with down-regulated P2X7 demonstrated significantly lower calcium signal in comparison to the control cells (Fig. 1b). We also estimated whether P2X7 activation may be responsible for ATP release from glioma C6 cells in the culture medium. BzATP (100 μM) treatment for 24 h increased the ATP release by 85% compared to the control. At the same time, BBG (100 nM) caused a notable decrease of ATP level in cell culture-conditioned medium from 185% after BzATP stimulation to 115% upon BzATP and BBG co-treatment for 24 h (Fig. 1c). All in all, P2X7 receptor activation is responsible for Ca^2+^ signal and ATP release in C6 glioma cells.

#### Activation of P2X7 increases C6 cell proliferation, viability, and cell adhesion* in vitro*

It is known that activation of P2X7 receptor increases the proliferation of ovarian carcinoma cells [38], lymphoid cells [39], neuroblastoma cells [40], etc. Similarly, we observed the stimulant effect of P2X7 activation on glioma C6 cell proliferation and viability.

**Fig. 2:**
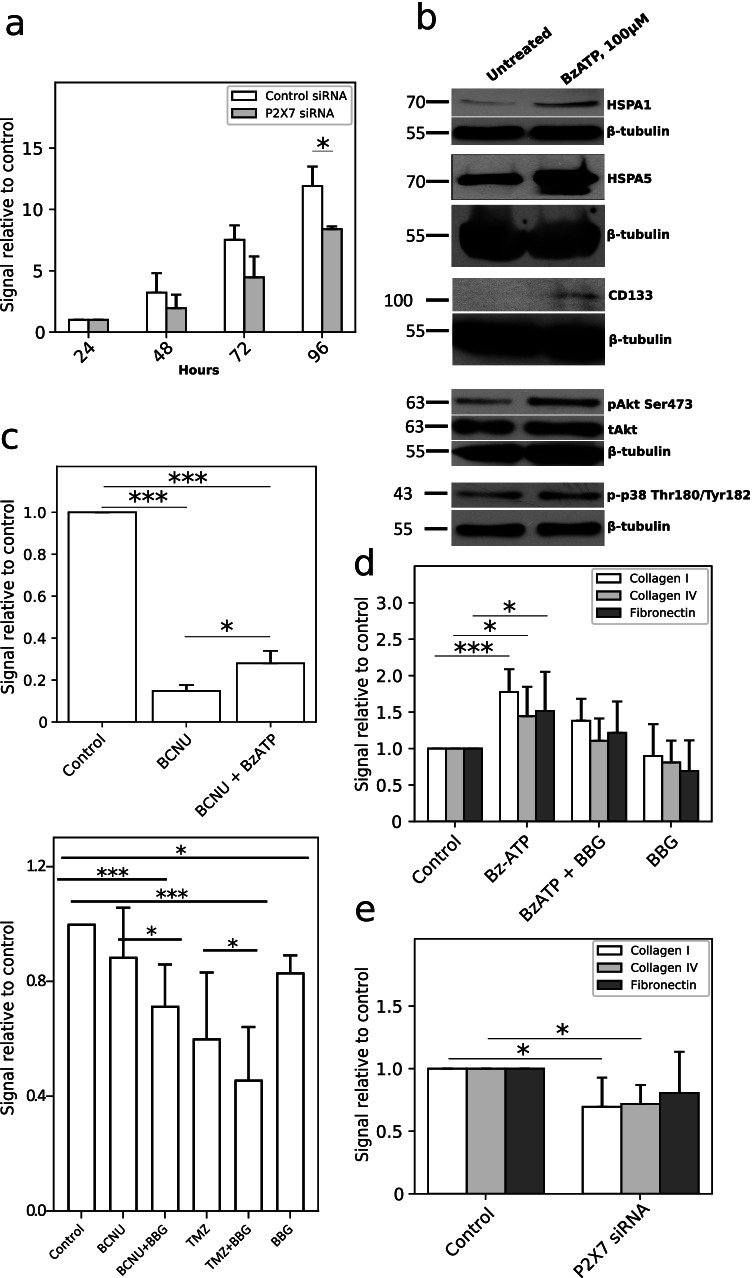
P2X7 activation increased C6 cell proliferation, viability, and cell adhesion in vitro. Quantitative data are presented as signal relative to control. **a** MTS test of long-term C6 cell viability after 100 µM BzATP stimulation of control cells and cells with P2X7 downregulation using RNAi (*n* = 3). The significance of the differences was determined with paired *t*-test: *P ≤ 0.05, **P ≤ 0.01, ***P ≤ 0.001 vs. the respective control. **b** Representative Western blot analysis of pro-survival and pro-proliferative proteins: p-p38 (Thr180/Tyr182), pAkt (Ser473), tAkt, CD133, HSPA1, and HSPA5 in C6 cell lysates after 24-h BzATP stimulation (*n* = 3). **c** Upper panel: MTS test of C6 cell viability after 200 µM BCNU (carmustine) treatment for 24 h. Co-treatment with 100 µM BzATP and 200 µM BCNU increased viability of C6 cells (*n* = 3). The significance of the differences was determined with one-way ANOVA with Bonferroni post hoc test: *P ≤ 0.05, **P ≤ 0.01, ***P ≤ 0.001 vs. all groups. Bottom panel: a crystal violet assay demonstrating a synergistic effect of BBG with BCNU/TMZ co-treatment on C6 cell growth for 24 h (*n* = 6). The significance of the differences was determined with repeated measures one-way ANOVA with Duncan’s multiple range post hoc test: *P ≤ 0.05, **P ≤ 0.01, ***P ≤ 0.001 vs. all groups. **d** P2X7 activation affects C6 cell adhesion to extracellular matrix components: collagen I, collagen IV, fibronectin. C6 cells showed higher adhesion potential after 24-h BzATP stimulation. 24-h co-treatment/treatment with 100 nM BBG significantly decreased C6 cell adhesion to ECM components (*n* = 6). The significance of the differences was determined with one-way ANOVA with Bonferroni post hoc test: *P ≤ 0.05, **P ≤ 0.01, ***P ≤ 0.001 vs. control group. **e** P2X7 RNAi downregulation declined C6 cell adhesion to ECM components upon BzATP stimulation (*n* = 4). The significance of the differences was determined with paired *t*-test: *P ≤ 0.05, **P ≤ 0.01, ***P ≤ 0.001 vs. the respective control

MTS test showed that RNAi-mediated downregulation of P2X7 expression led to the decrease of C6 cell viability compared to control cells treated with control RNAi. Cell viability was 60% lower after 48 h, 59% after 72 h, and 70% after 96 h of P2X7 knockdown (Fig. 2a). P2X7 influence on glioma cell proliferation was confirmed by Western-blot analysis of pro-survival and pro-proliferative proteins. We observed considerable increase of phosphorylated forms of p38 MAPK (Thr180/Tyr182) and Akt (Ser473) proteins, and an increase of CD133, HSPA1, and HSPA5 protein level in C6 cells stimulated with BzATP for 24 h (Fig. 2b). Interestingly, activation of P2X7 promoted C6 cell viability after treatment with chemotherapeutic drug. BzATP stimulation increased C6 cell viability by 13% after 200 μM of co-treatment with carmustine (BCNU) (Fig. 2c) in comparison to carmustine treatment only. We examined also whether inhibition of P2X7 potentiated the anticancer drug temozolomide (TMZ) and BCNU activity toward C6 cells. Cells were treated with 100 nM BBG and 600 μM TMZ or 200 μM of BCNU [41] only and in combination BBG + BCNU and BBG + TMZ for 24 h. We observed a statistically significant decrease of C6 cell growth after treatment with BBG + BCNU and BBG + TMZ compared to treatment with BCNU or TMZ only as well as compared to control untreated cells. P2X7 also had a positive influence on cell adhesion to the extracellular matrix components. To characterize the adhesion of C6 cells after P2X7 activation and inhibition, we performed cell adhesion assay to collagen I, collagen IV, and fibronectin. Activation of P2X7 significantly enhanced C6 cells adhesion to collagen I, collagen IV, and fibronectin. On the other hand, the treatment with 100 nM BBG decreased cell adhesion to all studied ECM components (differences were statistically non-significant) (Fig. 2d). To confirm the P2X7 influence on cell adhesion, C6 cells with P2X7 RNAi silencing were used. P2X7 downregulation suppressed C6 cell adhesion to both collagen I and IV, and to fibronectin (Fig. 2e). In short, activation of P2X7 boosted C6 cells proliferation and increased cell viability even under the condition of anticancer drug treatment. Moreover, BBG in combination with chemotherapeutics demonstrated a synergistic effect in the inhibiting of C6 cell growth.

### P2X7 effect on ROS production and mitochondrial membrane potential in glioma C6 cells

**Fig. 3:**
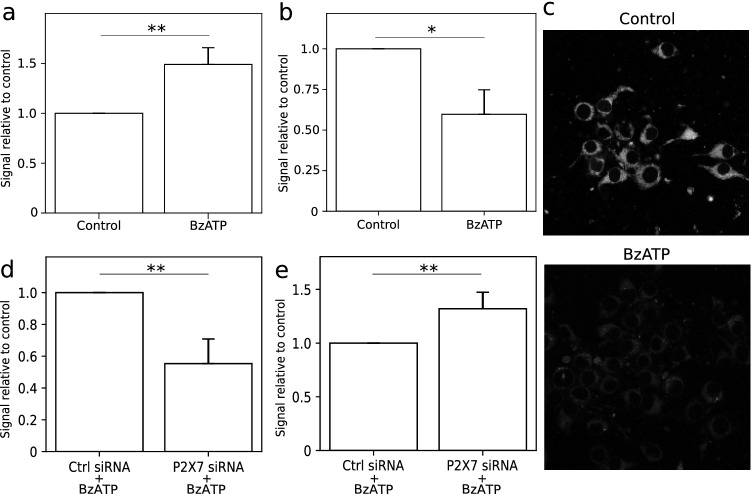
Activation of P2X7 receptor led to elevated ROS production and mitochondrial membrane depolarization in C6 cells. Quantitative data are presented as signal relative to control. **a** DCF-DA fluorescence measurement in C6 glioma cells. Cells stimulated with 100 µM BzATP for 1 h were characterized by increased ROS production compared to control in C6 cells (*n* = 3). The significance of the differences was determined with one-way ANOVA with Bonferroni post hoc test: *P ≤ 0.05, **P ≤ 0.01, ***P ≤ 0.001 vs. control group. **b** Potential-dependent staining of mitochondria in C6 cells using JC-1. 1-h BzATP (100 µM) stimulation led to considerable mitochondrial membrane depolarization (*n* = 3). The significance of the differences was determined with one-way ANOVA with Bonferroni post hoc test: *P ≤ 0.05, **P ≤ 0.01, ***P ≤ 0.001 vs. control group. **c** Representative images of MitoTracker Red CMXRos accumulation in C6 cells stimulated with 100 µM BzATP for 1 h. **d** DCF-DA fluorescence measurement in C6 glioma cells with RNA interference of P2X7 expression (*n* = 4). Downregulation of P2X7 led to decreased ROS production in C6 cells after 1-h BzATP (100 µM) stimulation. The significance of the differences was determined using paired *t*-test: *P ≤ 0.05, **P ≤ 0.01, ***P ≤ 0.001 vs. the respective control. **e** Potential-dependent staining of mitochondria using JC-1 dye in C6 cells with RNA interference of P2X7 expression after 1 h of 100 μM BzATP pre-treatment (*n* = 3). The amount of depolarized mitochondria was higher in control cells when compared to that in cells with P2X7 downregulation. The significance of the differences was determined using paired *t*-test: *P ≤ 0.05, **P ≤ 0.01, ***P ≤ 0.001 vs. the respective control

BzATP stimulation led to elevated ROS production in C6 cells. Using DCF-DA probe, we observed 57% increase of ROS production in cells stimulated by BzATP for 1 h compared to control cells (Fig. 3a). Activation of P2X7 also resulted in mitochondria depolarization. Using JC-1 probe, we established that 40% of mitochondria was depolarized in C6 cells stimulated with 100 µM BzATP for 1 h (Fig. 3b). C6 cells stimulated with 100 µM BzATP for 1 h also demonstrated a lower MitoTracker Red CMXRos accumulation in mitochondria (Fig. 3c) which confirmed mitochondrial membrane depolarization after P2X7R activation. We also used specific RNAi for inhibition of P2X7 receptor gene expression to verify the results from ROS measurement and mitochondrial membrane potential evaluation. We showed that ROS production in cells with P2X7 downregulation was 55% lower compared to control cells (Fig. 3d) and the mitochondria depolarization was 32% lower compared to control cells (Fig. 3e) after 100 µM BzATP treatment for 24 h, which was proportional to the protein level drop. Activated P2X7 is responsible for ROS overproduction and mitochondrial membrane depolarization in C6 cells.

#### P2X7 inhibition suppresses tumor growth and expansion *in vivo*

**Fig. 4:**
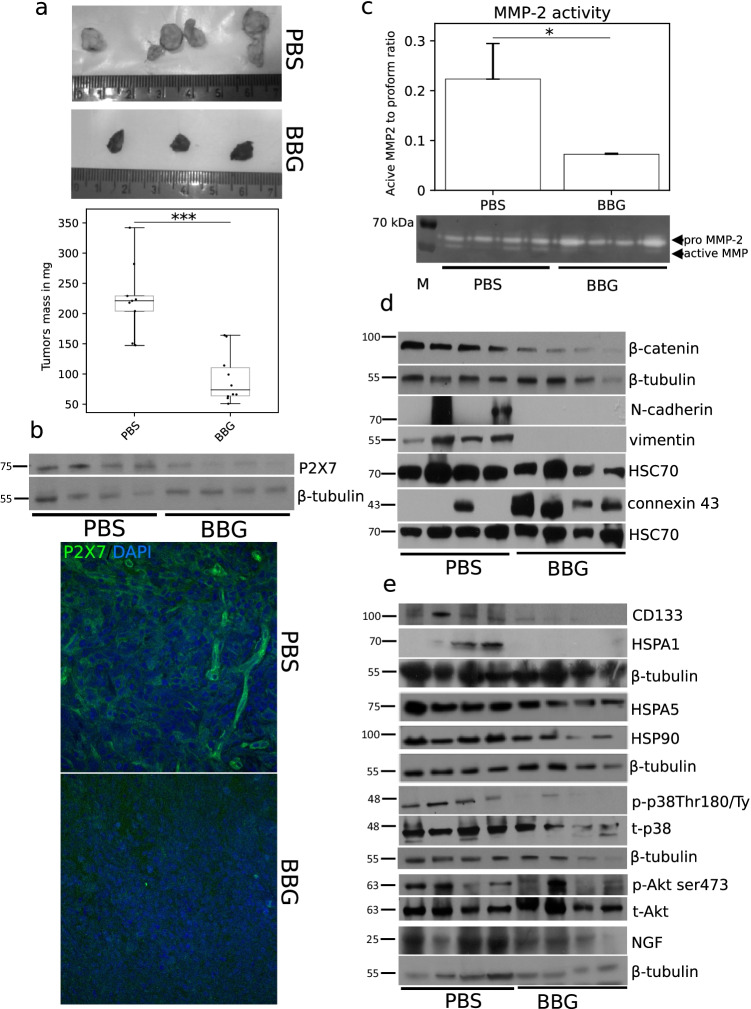
Effect of P2X7 inhibition using BBG on glioma C6 tumor development in vivo. **a** Brilliant Blue G administration led to reduction of glioma tumor mass. Representative images of control and BBG-treated C6 glioma tumors (*n* = 9 for control group and *n* = 10 for BBG-treated group). The significance of the differences was determined using Student’s *t*-test: *P ≤ 0.05, **P ≤ 0.01, ***P ≤ 0.001 vs. the respective control. **b** Representative Western blot showing the significant decrease of P2X7 expression in tumor homogenates after BBG treatment. Representative image of P2X7 immunodetection in C6 glioma tumors. **c** P2X7 inhibition resulted in diminished activation of matrix metalloproteinase-2 in glioma tumors. The significance of the differences was determined with Student’s *t*-test: *P ≤ 0.05, **P ≤ 0.01, ***P ≤ 0.001 vs. the respective control. **d** Western blot analysis of known proteins involved in cell adhesion and EMT signaling in C6 tumor homogenates (*n* = 4). **e** Western blot analysis of known proteins involved in tumor aggressiveness in C6 tumor homogenates (*n* = 4)

**Fig. 5:**
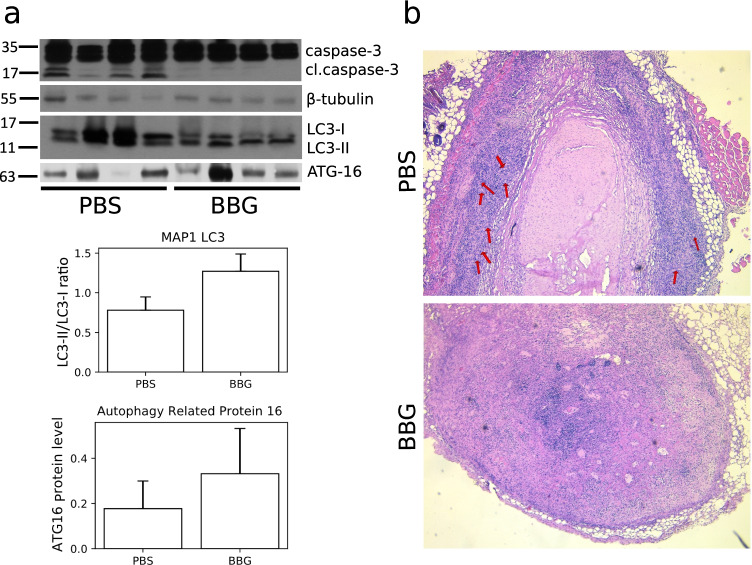
**a** Representative Western blot showing decline of caspase-3 cleavage and changes in the level of autophagy-related proteins in tumor homogenates after BBG treatment. The densitometric analysis plots of LC3 and ATG16 proteins placed below the Western blot image. **b** Representative image of hematoxylin and eosin staining of C6 glioma tumors. The images were acquired using × 20 objective. Arrows point to palisades and pseudopalisades in the tumor tissue

**Fig. 6:**
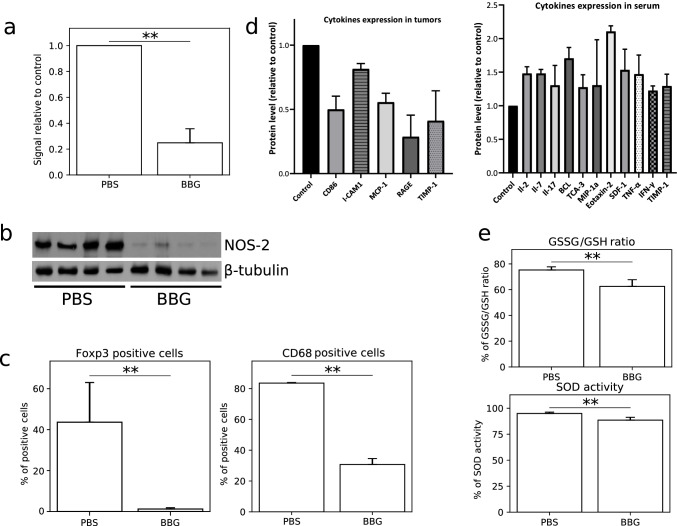
P2X7 inhibition reduced the ATP release with simultaneous decrease of some pro-inflammatory cytokine levels in mouse serum and changed the redox metabolism in C6 tumors. **a** An ATP release is significantly lower in tumors treated with BBG compared to the control tumors (*n* = 4). The significance of the differences was determined using Student’s *t*-test: *P ≤ 0.05, **P ≤ 0.01, ***P ≤ 0.001 vs. the respective control. **b** Decline NOS-2 expression in BBG-treated tumors (*n* = 4). **c** Decreased expression of FOXP3 (T_reg_ lymphocytes) and CD68 (macrophages) markers in BBG-treated tumors evaluated using flow cytometry (*n* = 5). The significance of the differences was determined using Student’s *t*-test: *P ≤ 0.05, **P ≤ 0.01, ***P ≤ 0.001 vs. the respective control. **d** The level of the pro-inflammatory and anti-inflammatory cytokines, in mouse serum and glioma tumors using cytokine array for multiplex protein detection (*n* = 3). **e** P2X7 inhibition caused a decrease of GSSG/GSH ratio with a slight decline of SOD activity in BBG-treated tumors (*n* = 4). The significance of the differences was determined using Student’s *t*-test: *P ≤ 0.05, **P ≤ 0.01, ***P ≤ 0.001 vs. the respective control

To confirm the influence of P2X7 receptor on C6 tumor growth, an in vivo experiment was performed. Administration of BBG led to a significant reduction in tumor mass from 214.7 ± 60.8 mg in the control group of mice to 92.6 ± 39.5 mg in mice treated with BBG (Fig. 4a). The level of P2X7 protein was significantly reduced in tumors treated with BBG compared to control tumors (Fig. 4b). BBG treatment also led to a 3.14-fold decrease of MMP-2 activity in the examined tumors as compared to control (Figs. 4c and 5) and these results correlated with a 29% increase of TIMP1 (TIMP metalloproteinase inhibitor 1) level in serum of BBG-treated mice (Fig. 6d) in comparison to the control. We also analyzed the level of proteins responsible for cell adhesion and migration in the studied tumors. BBG treatment decreased β-catenin level in the tumors. Interestingly, we also noticed a vast reduction of vimentin and N-cadherin (Fig. 4d) protein expression as well as decrease of ICAM-1 (Fig. 6d), but an increase of gap junction protein connexin 43 level in tumors after BBG treatment (Fig. 4d). P2X7 inhibition suppressed the expression of pro-survival proteins in tumors: CD133; neuronal growth factor (NGF); and chaperones HSPA1, HSPA5, and HSP90. We also observed a decrease of Akt (Ser473) and p38MAPK (Thr180/Tyr182) phosphorylation in BBG-treated tumors as compared to the control tumors (Fig. 4e). Based on these data, we suggest that inhibition of P2X7 receptor suppresses tumor growth and spreading in vivo.

The BBG treatment led to a decrease of tumor mass but caspase-3, a crucial executor of apoptosis, was not activated in treated tumors (Fig. 5a). Therefore, we also examined the expression of autophagy markers LC3-I, LC3-II, and Atg16 in studied tumors using Western blot. We found that P2X7 inhibition led to an increase of Atg16, LC3-II expression, and LC3‐II/LC3‐I ratio, in treated tumor tissues compared to control samples (Fig. 5a). That indicated that autophagic cell death occurred after BBG treatment. The densitometric analysis of Western blots of relative protein levels for Figs. 4b, d, and e and 5a is presented in Supplementary Figs. S2 and S3. The pathological changes in frozen tumors were investigated on hematoxylin and eosin-stained sections. We observed a large necrotic zone in the core mass of untreated tumor which was surrounded by palisading and pseudopalisading cells (Fig. 5b). In contrast to control, we did not observe pseudopalisading necrosis in BBG-treated tumors. The clusters of living cells, as well as small necrotic areas, were localized diffusive in the different parts of these tumors (Fig. 5b). Based on these data, we suggest that inhibition of P2X7 receptor suppresses tumor growth and spreading in vivo.

#### P2X7 inhibition alters immune-cancer interaction and redox status of tumors

In order to investigate a systemic immune response after P2X7 inhibition, we first evaluated the level of ATP in tumor microenvironment. It is widely known that extracellular ATP stimulates P2 receptors resulting in development of inflammation [42]. During carcinogenesis, ATP is released from dying cells continuously. We noticed a 65.6% reduction of ATP level in tumors after BBG treatment (Fig. 6a). We also observed that the level of FOXP3 (T_reg_ lymphocyte marker) and CD68 (PBMC marker) was significantly lower in BBG-treated tumors as compared to control tumors (Fig. 6c). P2X7 inhibition also reduced the level of receptor for advanced glycation end products (RAGE), MCP-1, and CD86 proteins in treated tumors (Fig. 6d). We then analyzed the pro-inflammatory and anti-inflammatory cytokines in mice serum using cytokine array for multiplex protein detection. The level of IL-2, IL-7, IL-17, BLC, TCA-3, MIP-1α, Eotaxin-2, SDF, TNFα, and IFNγ was statistically higher in serum of BBG-treated mice as compared to control mice (Fig. 6d). Inhibition of P2X7 receptor led to the changes in redox metabolism in C6 tumors. We analyzed the ratio between disulfide-oxidized glutathione level (GSSG) and reduced glutathione (GSH) in the studied tumors. P2X7 inhibition resulted in a decrease of GSSG/GSH ratio from 74.5% in control tumor homogenates to 66.3% in the tumor homogenates from the treated group (Fig. 6e). We also noted a slight decline (5%) in the activity of SOD (Fig. 6e). Moreover, the level of NOS-2 was decreased in BBG-treated tumors as compared to control tumors (Fig. 6b). Taken together, this suggests that P2X7 inhibition modulates the tumor microenvironment via a decrease in ATP release, alteration of some immune cells markers in the tumor mass, and changes in the redox metabolism in C6 tumors.

#### Activity of P2X7 receptor in human gliomas

**Fig. 7:**
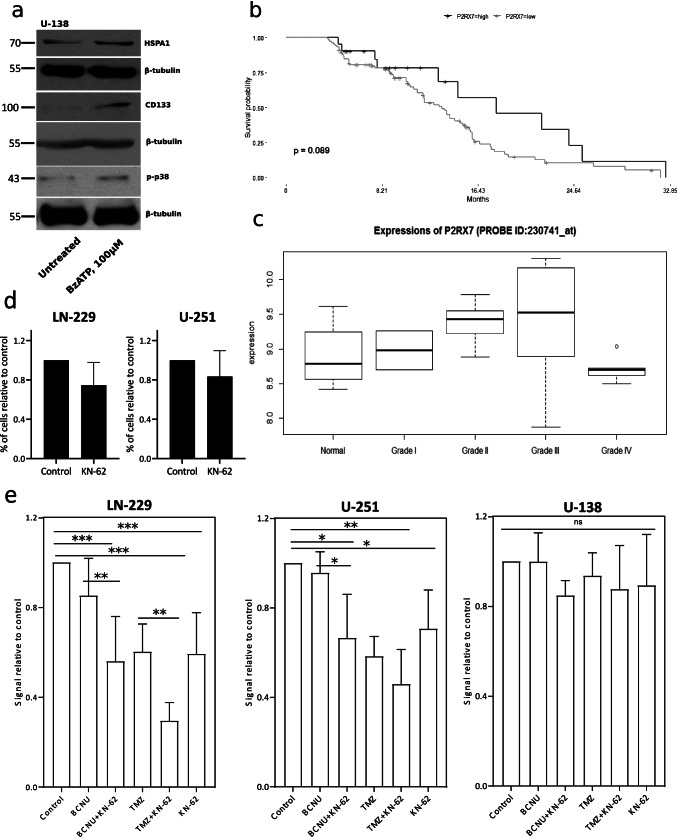
P2X7 receptor effects on human glioma development. **a** Western blot analysis of p-p38 MAPK, CD-133 and HSPA1 expression in U-138 human glioma cells (*n* = 3). **b** In silico analysis of P2X7 expression level and survival of glioma patients using information from the TCGA database. **c** In silico analysis of P2X7 expression level in different WHO astrocytoma grades compared to normal tissue using NCBI dataset. **d** A transwell migration assay of LN-229 and U-251 glioma cells treated with 2 μM KN-62 for 24 h (*n* = 3). **e** A crystal violet assay demonstrating a synergistic effect of 2 μM KN-62 with BCNU/TMZ co-treatment on LN-229, U-251, and U-138 glioma cell growth for 24 h (*n* = 4). The significance of the differences was determined with repeated-measures one-way ANOVA with Duncan multiple range post hoc test: *P ≤ 0.05, **P ≤ 0.01, ***P ≤ 0.001 vs. all groups

Human glioma cell lines U-251 and U-138 are characterized by a low P2X7 expression when compared to rat glioma C6 cells, as was described previously [43]. Nevertheless, we observed an increase of p38 MAPK phosphorylation and increase of HSPA1 and CD133 protein level in U-138 glioma cells stimulated with 100 μM of BzATP for 24 h (Fig. 7a). The densitometric analysis of Western blots of relative protein level for Fig. 7a is presented in Supplementary Fig. S2. To estimate the dependence between P2X7 expression and the survival of glioma patients, we analyzed a cohort of 131 patients using information from TCGA database. We did not find any correlation between P2X7 expression level and survival of glioma patients (Fig. 7b). However, this database does not allow comparing the P2X7 expression level between non-malignant and glioma tumor tissues. Next, we used a NCBI dataset from a study which utilized a pathway-specific enrichment analysis of the microarray data and quantified the gene expression difference between astrocytoma tumors and pooled normal tissues [44]. The authors analyzed the expression of 54,676 probe IDs using Affymetrix HU-133PLUS 2.0 GeneChip microarrays for 15 astrocytoma tissue samples: grade II, III, and IV tumors, five samples per grade; and 4 normal tissue specimens [45]. We found P2X7 receptor upregulation in tumors of WHO grades II and III compared to normal tissue and downregulation of the receptor in tumors of WHO grade IV (Fig. 7c). In the same database, we looked for genes we identified as upregulated in glioma C6 cells stimulated with BzATP. The database also showed an upregulation of caspase-3, Akt, N-cadherin, MMP-2, HSPA1, and p38 MAPK α subunit in grades II, III, and IV compared to the normal tissue (Supplementary Fig. S1). We also examined human glioma cell invasiveness through matrigel-coated transwell chambers. LN-229 and U-251 cell suspensions were mixed with 2 μM KN-62 and placed in the upper chamber. We noticed a 25% decrease of LN-229 cell invasion and a 17% decrease of U-251 cells, but these data are statistically non-significant (Fig. 7d). However, KN-62-mediated P2X7 antagonism potentiated a cytotoxic activity of anticancer drugs BCNU and TMZ compared to KN-62 treatment only (Fig. 7e). Such dependence was statistically significant in the case of LN-229 and U-251 cells, similar to C6 cells and non-significant for U-138 cells. On the whole, increased expression of P2X7 in human glioma samples suggests that this receptor is rather a negative prognostic marker for glioma patients.

## Discussion

The glioma tumor microenvironment is characterized by an abundant amount of ATP released from active neurons [46] as well as dying or stressed cells and cells after chemotherapy [47, 48]. Extracellular ATP activates metabotropic P2Y receptors and ionotropic P2X receptors. In particular, a high concentration of ATP leads to P2X7 receptor activation [4], which is present on glioma cells as well as microglial cells [47]. Summarizing the literature data, the role of P2X7 receptor in glioma development is ambiguous. There are reports on cell death induction via P2X7 activation in cancer cells. For example, activation of P2X7 diminished the cell viability of radiosensitive M059J glioma cells [49]. P2X7 agonists also induced cell death in the GL261 mouse glioma [50]. On the other hand, we found even more information about P2X7 promoting glioma tumor growth. For instance, it was shown that activation of P2X7 receptor did not induce cell death in C6 glioma cells and, on the contrary, increased the tumor growth [23, 51]. In the other report, authors demonstrated that apyrase, an ATP-hydrolyzing enzyme, reduces glioma tumor growth and proliferation [25]. Nevertheless, the role of the P2X7 receptor in glioma development is still poorly characterized and requires a more detailed study of signaling pathways associated with P2X7 activation/inhibition in glioma cells in vitro and the influence of receptor’s activity on the tumor microenvironment.

In the present study, we demonstrated pro-proliferative and pro-survival effects of P2X7 receptor activation in rat and human glioma models. The studied receptor was activated in vitro using a highly potent agonist of P2X7 and BzATP. Continuous activation of P2X7 by BzATP led to an increase of P2X7 protein level in rat C6 glioma cells. The activation also promoted the pore formation, evidenced by ethidium bromide uptake, and increased calcium signal in C6 glioma cells [43]. Also, P2X7 activation stimulated massive extracellular ATP release from C6 cells into the culture medium. Simultaneously, BzATP treatment did not result in cell death but boosted C6 cell proliferation and viability in vitro. The selective antagonist of rat’s P2X7 receptor, BBG, effectively reduced the effects of receptor activation and decreased C6 cell viability and ATP release. Moreover, the levels of pro-survival proteins CD133, HSPA1, and HSPA5 as well as phosphorylation of p38 MAPK and Akt were increased in cells treated with BzATP. A significant cytoprotective effect of BzATP was also found, manifested in lower cytotoxicity of carmustine toward C6 cells cultured in vitro. And the opposite effect was observed in the case of P2X7 antagonism and simultaneous treatment with chemotherapeutics. We observed a statistically significant decrease of C6 cell growth after BBG + BCNU and BBG + TMZ co-treatment compared to treatment with BCNU or TMZ only as well as compared to untreated cells. P2X7 activation increased ROS production and mitochondria depolarization. Importantly, increased ROS may play an important role in maintaining the cancer phenotype [52]. It was shown that cell adhesion to the vascular wall is enhanced by mitochondrial ROS production stimulated by G protein-coupled receptor-Ca^2+^ signaling [53]. We also studied the influence of P2X7 activation on C6 cell adhesion to collagens, the most abundant matrix protein polymers, which increase tumor tissue stiffness, regulate tumor immunity, and promote metastasis [54]. Moreover, the invasive phenotype of neoplastic cells is supported by secretion of extracellular matrix proteins (fibronectin, vitronectin, collagen) by GBM cells in an integrin-dependent manner [55]. We showed that cells treated with 100 μM BzATP demonstrated a significantly higher adhesion to collagen I, collagen IV, and fibronectin compared to untreated cells. All these data suggest that P2X7 activation can change the phenotype of glioma cells cultured in vitro more aggressively through enhanced cell proliferation and adhesion to ECM components and ATP release, and by increased expression of chaperones and kinases essential for cancer cell growth.

The influence of P2X7 receptor on C6 glioma tumor growth was also shown on the tumor model in vivo. P2X7 is expressed abundantly in microglia [56] and activation of this receptor in microglial cells might affect glioma tumor growth into the brain. To avoid the influence of microglial P2X7 activation on C6 tumor development, we decided to inject the cellular mass heterotopically, under the skin. Other [23, 57] authors started the administration of P2X7 inhibitor immediately after C6 cell injection or co-injected cells with inhibitor. Unlike them, we examined P2X7 antagonism effectiveness on well-developed tumors, and BBG administration was started 2 weeks after C6 cell inoculation. P2X7 inhibition by BBG significantly decreased the mass of formed C6 glioma tumors. Some controversial study showed a pro-proliferative and tumor growth stimulatory effect of BBG on C6 glioma cells [58]. Current investigation together with aforementioned studies showed an inhibitory effect of P2X7 antagonist on C6 glioma tumor growth in xeno- and allotransplantant models. The histological studies showed a significant difference between untreated and treated tumors. Control tumors were characterized by palisading and pseudopalisading structures, and a large necrotic zone. Pseudopalisading necrosis and vascular proliferation are the two important hallmarks of glioblastoma [59]. Since pseudopalisades are severely hypoxic, overexpress hypoxia-inducible factor (HIF-1), and secrete proangiogenic factors such as VEGF and IL-8, they induce a wave of tumor cells actively migrating away from the central hypoxic area [60]. The histopathological changes of BBG-treated tumors are represented by small necrotic areas diffused on the whole tumor without pseudopalisading cells. Tumors from BBG-treated mice were characterized by decreased AKT phosphorylation which is believed to suppress tumor cell survival [61, 62]. What is more, decreased expression of HSPA1, HSPA5, and HSP90 chaperones in BBG-treated tumors most probably deprived the cancer cells of the protective shield against the stressful factors in the tumor microenvironment, which promotes the elimination of cancer cells [63, 64]. P2X7 inhibition may have also affected tumor cells spreading as evidenced by a decreased activity of matrix metalloproteinase-2, an essential enzyme for extracellular matrix degradation [65]. At the same time, we observed an increase of TIMP metalloproteinase inhibitor 1 level in the serum of BBG-treated mice. Analogous results showed that neuroblastoma cells lack the ability to infiltrate lung tissue and bone marrow of immunodeficient mice under BBG treatment [66]. Upon the receptor inhibition, we also observed the decreased expression of CD133 protein which is involved in glioma tumor cell invasiveness [67] and attributes to glioma stem cell marker appearance [68]. The p38-MAPK (both phosphorylated form and total protein) level was decreased in tumors after BBG treatment. P2X7 inhibition also decreased the level of β-catenin, an epithelial-mesenchymal transition (EMT)-related protein [69]. Interestingly, expression of negative prognostic cancer markers [70, 71], N-cadherin and vimentin, was strongly reduced in BBG-treated tumors. Other authors showed that P2X7 activation considerably increased C6 cell mobility in vitro which was largely inhibited in the presence of P2X7 antagonist [51]. These results are in line with our data, obtained from tumors in vivo. All these data testify to the decline of C6 glioma cell migration ability after P2X7 receptor inhibition.

Purinergic receptors participate in inflammation and immunity in normal [8, 72] and cancer tissues [73]. In particular, P2X7 receptor, activated by extracellular ATP, facilitates ATP-dependent NLRP3 inflammasome formation and IL-1β release in head and neck squamous carcinoma [74]. The high availability of extracellular ATP in tumor mass leads to the continuous activation of P2X7 receptor. Subsequently, the successive ATP release into the extracellular space creates a “vicious circle” of repeated activation of P2X7 and the promotion of tumor growth [24]. We observed a significant decrease in the ATP amount in tumor samples after BBG inhibition of the P2X7 receptor. The simultaneous decrease of tumor mass confirms the importance of the P2X7 activation for glioma development in vivo. Another interesting effect of the receptor inhibition was a total reduction of cleaved caspase-3 amount in tumors after BBG treatment. It is known that caspase-3 activation is linked to increased cancer cell migration and development of inflammation in gliomas [75]. The loss of tumor mass after BBG treatment can be explained by the activation of autophagy in glioma cells. We observed an increased expression of essential proteins for autophagosome formation: ATG-16 [76] and LC3-II [77] after P2X7 inhibition.

A number of studies confirmed the stimulant effect of microglia and infiltrated macrophages on glioma progression [78, 79]. ATP, together with chemokines, can modulate tumor microenvironment shape acting as chemoattractant for tumor-associated macrophages [80]. Based on that information and our results, we suggest that P2X7 receptor can affect the immune response during glioma development. We observed a significant decrease of CD86 (b7-2) and CD68 marker level in BBG-treated tumors. Upregulation of CD68 is associated with a higher malignancy in gliomas and poor prognosis for glioma patients [81]. The P2X7 inhibition led to the decline of FOXP3^+^ T_reg_ lymphocyte marker level. Fang KM et al. showed previously that P2X7 inhibition by oxATP administration decreased the expression of MIP-1α and MCP-1 in the C6 tumors as well as reduced the number of microglia/macrophages infiltrating tumor [57]. These results are in line with our investigations, whereas BBG administration led to downregulation of CD86, I-CAM1, MCP-1, RAGE, and TIMP-1 in C6 tumors. P2X7 receptor also modulated the profile of produced cytokines in the peripheral blood serum. We analyzed the expression of 44 cytokines and found that the amount of IL-2, IL-7, IL-17, BLC, TCA-3, MIP-1α, Eotaxin-2, SDF, TNFα, and IFNγ was significantly higher in mouse serum treated with BBG. Additionally, we observed a decreased amount of nitric oxide synthase 2 in BBG-treated tumor mass in comparison to the control tumors. Moreover, the P2X7 inhibition resulted in a shift of GSSG/GSH ratio toward glutathione-reduced state and a slight decline of SOD activity compared to the control tumors. Taken together, P2X7 seems to play an active role in the formation of inflammatory microenvironment promoting cancer progression.

The recent studies showed that antagonism of P2X7 receptor suppressed human glioma cell proliferation in vitro in U-251 human model glioma cells and in primary cell culture obtained from human glioma samples. The same authors demonstrated decreased expression of several pro-inflammatory factors in human glioma cells [82, 83]. In our previous study, we did not observe P2X7 stimulant effect on human U-251 and U-138 glioma cell proliferation. Moreover, P2X7 receptor seemed to be inactive in U-138 glioma cells [43]. In the present study, we showed the stimulant effect of P2X7 on p38 MAPK phosphorylation and CD133 and HSPA1 expression in U-138 cells. On the other hand, Bergamin et al. showed that transfection of these cells with P2X7 increased U-138 growth in vivo but not in vitro indicating the supporting role of P2X7 in glioma development [84]. Additionally, inhibition of P2X7 in LN-229 and U-251 cells decreases aggressiveness in vitro. Since anticancer chemotherapy induced massive ATP release, which activated purinergic receptors [48], the inhibition of P2X7 can be considered concomitant therapy of glioma. Gehring MP et al. showed that P2X7 activation increased effectiveness of radiotherapy using GL261 and M059J cell lines which were sensitive to ATP-P2X7R-induced cell cytotoxicity [85], whereas we performed our studies using LN-229, U-251, and U-138 glioma cells, in which P2X7 did not induce cell death. Our data demonstrated a synergistic effect of anticancer drugs in combination with KN-62 on LN-229 and U-251 cell growth in vitro. We also performed a meta-analysis of P2X7 gene expression using data from TCGA-GBM biopsies and did not find any correlation between P2X7 expression level in tumor samples and the life expectancy of glioma patients. We explain this finding by the fact that TCGA database does not allow comparing the P2X7 expression level in non-malignant and glioma tumor tissues. Analyzing gene expression data from the NCBI dataset, we revealed a P2X7 receptor upregulation in tumors of WHO grades II and III in comparison to the normal tissue. Ji Z et al. showed an increased P2X7 expression in human glioma samples of grade IV compared to grades I–III [86]. These data are in contradiction with our results of microarray analysis of astrocytoma samples. Here, we show that expression of P2X7 is downregulated in the grade IV compared to grade I–III and normal tissues. Moreover, J. Liu et al. showed a decrease of P2X7 level in GBM samples compared to normal peripheral brain tissue. The same authors explained this phenomenon by hypermethylation of P2X7R promoter in glioma samples [87]. In the same tumor samples, we observed an upregulation of caspase-3, Akt, N-cadherin, MMP-2, HSPA1, and p38 MAPK α subunit in grades II, III, and IV compared to normal tissue. These data are in line with our current results obtained from C6 rat glioma model in vivo.

## Conclusions

To summarize the in vitro data, BzATP activation of nucleotide P2X7 receptor induced a massive ATP release together with an upregulation of P2X7 expression and an increase of calcium signal in glioma C6 cells. Nevertheless, P2X7 activation did not induce cell death in the studied cells. Conversely, the cells demonstrated increased viability and cell adhesion to collagens after the receptor stimulation. Additionally, the level of p38 MAPK and Akt phosphorylation was higher after P2X7 activation, similarly to the total protein level of chaperones HSPA1 and HSPA5, and glioma stem cell marker CD133. Also, activated P2X7 receptor led to mitochondria depolarization and increased ROS production in glioma C6 cells. A comparable effect of P2X7 influence on glioma tumor development was observed in vivo: P2X7 receptor inhibition by Brilliant Blue G caused a significant decrease of tumor mass, a downregulation of P2X7 expression, and considerable decrease of ATP amount inside the tumors. P2X7 inhibition may have affected glioma tumor cells spreading as evidenced by a decrease of matrix metalloproteinase-2 activity and decrease (β-catenin) or total reduction (N-cadherin and vimentin) of EMT-related proteins. Such short-term therapy influenced the antioxidant defense system and modulated an immune response toward the glioma tumor. Based on the results, we propose that P2X7 receptor influences glioma cell survival and spreading in vitro and in vivo. P2X7 inhibition in vitro and in vivo decreased the expression of some negative prognostic cancer markers: pro-survival proteins, EMT-related proteins as well as decreased the ATP level, and ROS production in glioma C6 cells. Moreover, the expression of P2X7 was upregulated in the human astrocytomas at WHO grades II and III. All this taken together shows that P2X7 receptor supports glioma growth and spreading.

## Supplementary Information

Below is the link to the electronic supplementary material.

Supplementary file1 (DOCX 437 kb)

## Data Availability

The datasets generated and analyzed during the current study are available from the corresponding author on reasonable request.
