# Supplementary material for: P2X7 receptor: the regulator of glioma tumor development and survival
Source: Purinergic Signal. 2021 Dec 29;18(1):135–54. doi: 10.1007/s11302-021-09834-2 (PMC8850512; doi:10.1007/s11302-021-09834-2)
Supplement: Supplementary file 1 — Supplementary file1 (DOCX 437 kb) [file 11302_2021_9834_MOESM1_ESM.docx]

Supplementary Materials:

**P2X7 receptor: the regulator of glioma tumor development and survival**

Damian Matyśniak, Vira Chumak, Natalia Nowak, Artur Kukla, Liliia Lehka, Magdalena Oslislok and Paweł Pomorski


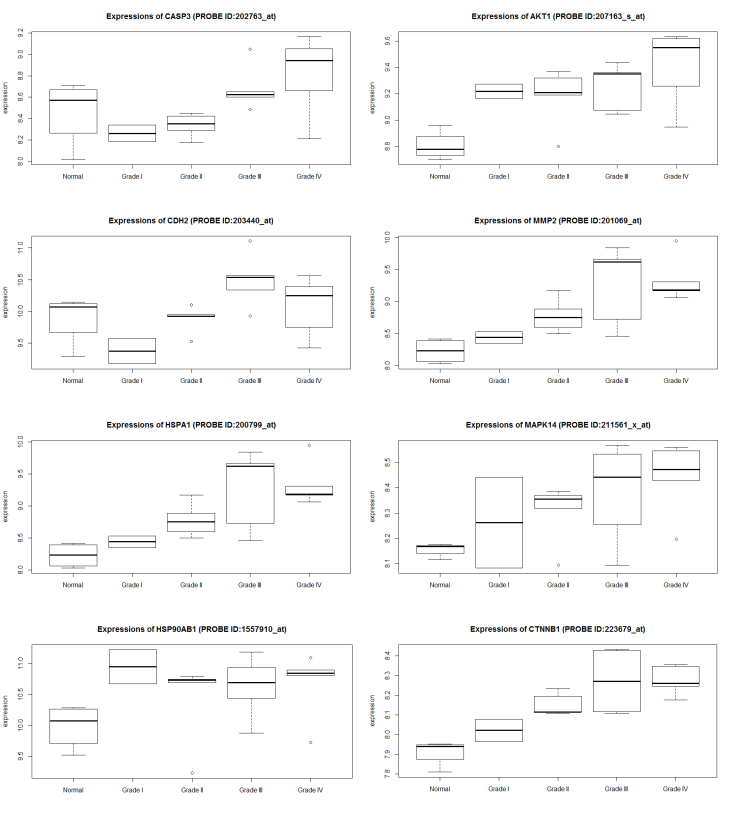
Figure S1. Level of Caspase-3, Akt kinase, N-cadherin, MMP-2, HSPA1, p38 MAPK α, HSP90 and β-catenin mRNA in grades II, III, and IV compared to normal tissue microarray.


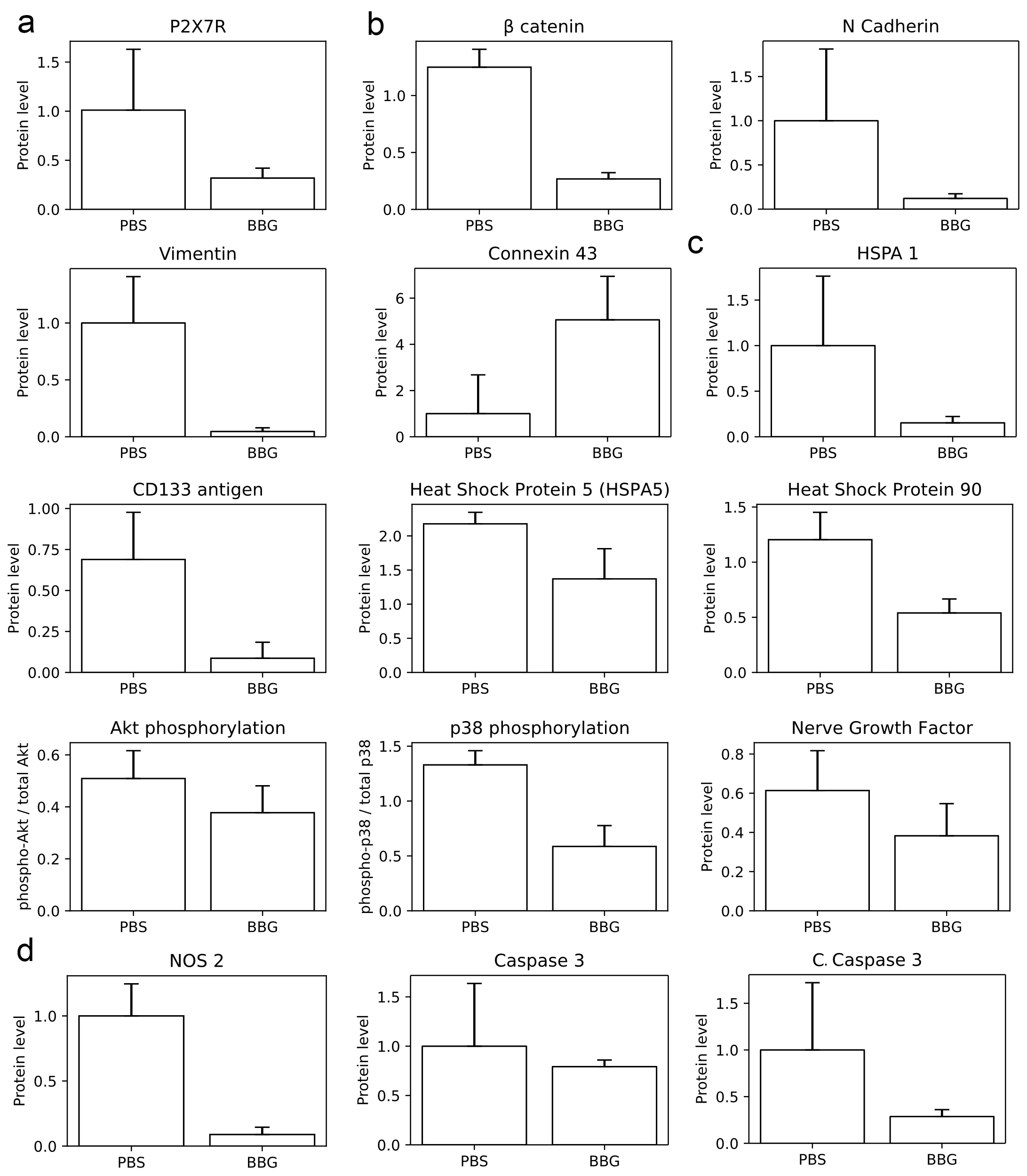
Figure S2. Semi-quantative analysis of Western blots performed using ImageJ program. (a) Densitometric analysis of relative protein level of P2X7 receptor (Figure 4B in manuscript). (b) Densitometric analysis of relative proteins level (Figure 4C in manuscript). (c) Densitometric analysis of relative proteins levels contained in figure 4D in manuscript. (d) Densitometric analysis of relative proteins levels provided in figure 5A and 6B in manuscript.


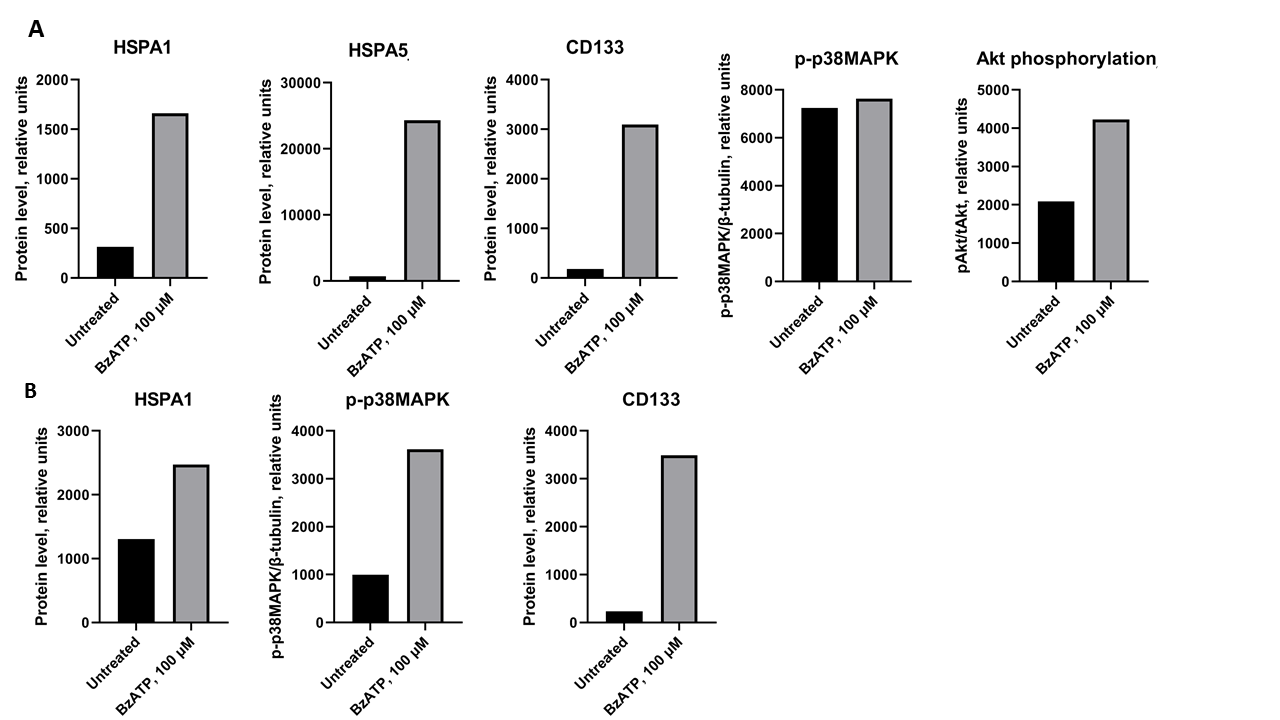
Figure S3. Semi-quantative analysis of Western blots performed using ImageJ program. (a) Densitometric analysis of relative proteins level (Figure 2B in manuscript). (b) Densitometric analysis of relative proteins level (Figure 7A in manuscript).

Table S1. Antibodies used in presented studies

| Antibody | Catalog number | Source | Application/dliution |
| --- | --- | --- | --- |
| Anti-β-Tubulin | T8328 | Mouse monoclonal | WB/1:10000 |
| Anti-β-catenin | sc-133240 | Mouse monoclonal | WB/1:1000 |
| Anti-CD133 (Prominin-1) | C9493 | Rabbit polyclonal | WB/1:100 |
| Anti-CD133 (Prominin-1) | ab19898 | Rabbit polyclonal | WB/1:100 |
| Anti-Caspase-3 | 9662 | Rabbit polyclonal | WB/1:1000 |
| Anti-connexin 43 | sc-13558 | Mouse monoclonal | WB/1:1000 |
| Anti-HSPA1 | ADI-SPA-810-F | Mouse monoclonal | WB/1:1000 |
| Anti-HSPA5/GRP78 | ab21685 | Rabbit polyclonal | WB/1:5000 |
| Anti-HSP90 | ADI-SPA-830 | Mouse monoclonal | WB/1:3000 |
| Anti-N-cadherin | sc-393933 | Mouse monoclonal | WB/1:1000 |
| Anti-NOS2 | sc-7271 | Mouse monoclonal | WB/1:1000 |
| Anti-HSC-70 | sc-7298 | Mouse monoclonal | WB/1:2000 |
| Anti-Phospho-Akt (Ser473) | 9271 | Rabbit polyclonal | WB/1:1000 |
| Anti-Akt | 9272 | Rabbit polyclonal | WB/1:1000 |
| Anti-Phospho-p38 MAPK (Thr180/Tyr182) | 9211 | Rabbit polyclonal | WB/1:1000 |
| Anti-p38 MAPK | 9212 | Rabbit polyclonal | WB/1:1000 |
| Anti-vimentin | sc-373717 | Mouse monoclonal | WB/1:2000 |
| Anti-β-Actin-HRP-conjugated | A3854 | Mouse monoclonal | WB/1:10000 |
| Anti-P2X7 | APR-004 | Rabbit polyclonal | WB/1:200 |
| Anti-NGF | ab68151 | Rabbit polyclonal | WB/1:500 |
| Anti-LC3A/B | 4108 | Rabbit polyclonal | WB/1:1000 |
| Anti-Atg16L1 | 8089 | Rabbit polyclonal | WB/1:1000 |
| APC anti-mouse CD68 Antibody | 137007 | Mouse monoclonal | FC/0.06 μg per million cells |
| APC Rat IgG2a, κ Isotype Ctrl Antibody | 400511 | Rat monoclonal | FC/0.06 μg per million cells |
| Alexa Fluor® 488 anti-mouse FOXP3 Antibody | 126405 | Mouse monoclonal | FC/0.25 µg per 106 cells |
| Alexa Fluor® 488 Rat IgG2b, κ Isotype Ctrl Antibody | 400625 | Rat monoclonal | FC/0.25 µg per 106 cells |
| Goat anti-Rabbit IgG (H+L) Highly Cross-Adsorbed Secondary Antibody, Alexa Fluor Plus 488 | A32731 | Goat polyclonal | IHC secondary antibody/ 1:200 |
| Goat Anti-Mouse IgG Antibody, HRP conjugate | 12-349 | Goat polyclonal | WB secondary antibody/1:10000 |
| Goat Anti-Rabbit IgG Antibody, HRP-conjugate | 12-348 | Goat polyclonal | WB secondary antibody/1:10000 |
